# Can choices between alternative hip prostheses be evidence based? a review of the economic evaluation literature

**DOI:** 10.1186/1478-7547-8-20

**Published:** 2010-10-29

**Authors:** Charlotte Davies, Paula Lorgelly, Ian Shemilt, Miranda Mugford, Keith Tucker, Alex MacGregor

**Affiliations:** 1 School of Medicine, Health Policy and Practice, University of East Anglia, UK; 2 Centre for Health Economics, Monash University, Australia; 3 Norfolk and Norwich University Hospitals, Colney Lane, Norwich, UK

## Abstract

**Background:**

Total hip replacement surgery places a considerable financial burden on health services and society. Given the large number of hip prostheses available to surgeons, reliable economic evidence is crucial to inform resource allocation decisions. This review summarises published economic evidence on alternative hip prostheses to examine the potential for the literature to inform resource allocation decisions in the UK.

**Methods:**

We searched nine medical and economics electronic databases. 3,270 studies were initially identified, 17 studies were included in the review. Studies were critically appraised using three separate guidelines.

**Results:**

Several methodological problems were identified including a lack of observed long term prosthesis survival data, limited up-to-date and UK based evidence and exclusion of patient and societal perspectives.

**Conclusions:**

More clinical trials including long term follow-up and economic evaluation are needed. These should compare the cost-effectiveness of different prostheses with longer-term follow-up and including a wider perspective.

## Background

About 8 million people in the UK have osteoarthritis (OA) [1]. Patients typically experience chronic pain and loss of physical function with an impact on society of lost productivity and increased burden on domiciliary/informal care. For those with end stage hip disease, total hip replacement (THR) surgery offers the only effective treatment. Over 70,000 THR operations were carried out in England and Wales in 2008/9 [2], with the number almost doubling in the last decade. As the population continues to age demand for this type of surgery will increase, with significant implications for the health system in terms of the impact on healthcare budgets and service utilisation. Inevitably, healthcare decision makers will need to make decisions that aim to ensure an efficient allocation of resources to THR surgeries, including the availability, timing and configuration of such interventions.

**Figure 1:**
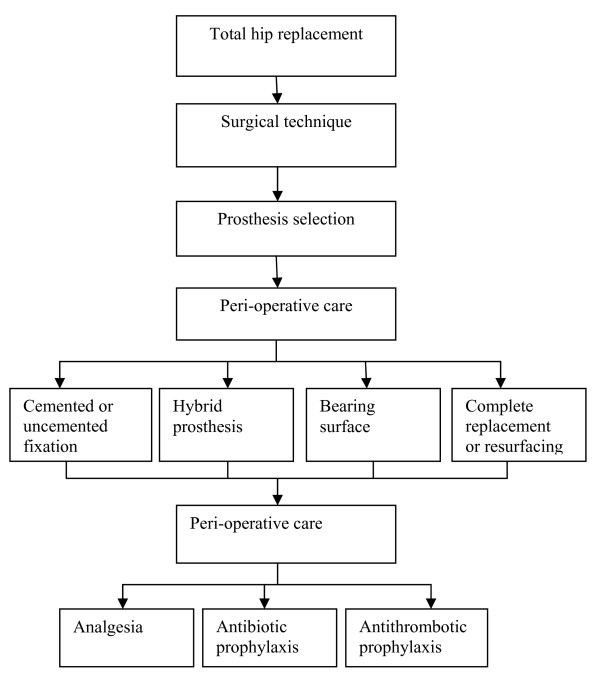
**Total hip replacement (adapted from 'map of medicine' - health guides, NHS Choices) **[44]. Treatment pathways available to those undergoing elective total hip replacement surgery in the NHS, UK.

The total cost of joint replacement surgery to the National Health Service (NHS), UK in 2000 was approximately £140 million [3], (£172 million in 2008 prices) [4], with the direct hospital costs of each procedure ranging from £488 to £9,905, mean of £4,788 [3](2008 prices). Predicted cost savings of total joint replacement surgery (relative to no surgery) are the reduced costs of arthritis treatment, medication and community care. In this paper we focus on total hip replacement surgery. Figure 1 illustrates the treatment pathways available to those undergoing elective THR surgery in the UK NHS.

In 1998 more than 60 hip prostheses manufactured by 19 companies were listed on the market in the UK [5], with total NHS expenditure on hip prostheses of approximately £53 million [3] (£67 million in 2008 prices). In 2008 the National Joint Registry (NJR) [2], listed 124 brands of acetabular cups and 137 brands of femoral stems, which indicates a substantial increase in the number of prostheses available from 1998 to 2008. In England and Wales, the National Institute for Health and Clinical Excellence (NICE) recognises three broad categories of prosthesis: cemented, cementless and hybrid [5]; with the NJR reporting an increased use of cementless procedures from 21% of all THR procedures recorded on the NJR in 2004 to 33% by 2008 [2].

THR is one of the most frequently performed surgical procedures in the world [6], with the average age of a patient receiving surgery reported as 66 years [7]. Revision surgery has increased with 3,012 revision procedures carried out in 2003/4, rising to 6,581 by 2008/9 [2,7] and accounting for approximately 9.4% of all THR procedures in England and Wales. Revision surgery is also a key element of cost with Briggs et al [8] reporting a mean cost for a standard hip or knee revision procedure in 2000/1 as £5,294 (£6,385; 2008 prices) compared to £3,889 (£4,690; 2008 prices) for a primary procedure. The prosthesis manufacturing industry has responded to the increase in demand for THR surgery by investing significant amounts of money in developing new, more durable, prostheses.

Economic evaluation is widely used to inform policy decisions regarding which new healthcare technologies should be adopted given the available resources [9]. NICE provides guidance to the NHS in England and Wales on clinical and cost-effectiveness of new and already developed technologies and within this, provides recommendations on the principles and methods of health technology appraisal [10].

From an economic perspective, some or all of the direct medical costs of implanting a new or alternative hip prosthesis may be offset by reductions in the subsequent direct medical costs associated with complications and/or secondary intervention and also by an earlier return to productive activity.

Health care purchasers (in the NHS, surgeons and clinical or finance managers) are motivated by a desire to buy the most effective prostheses for patients but are also constrained by health budgets, meaning they increasingly demand greater ‘value for money' from the prostheses. Potential important differences in non-medical resource use and costs may also result from the use of different prostheses. These include productivity losses (absence from paid/unpaid work) associated with differing lengths of rehabilitation/functional status; other patient out-of-pocket expenses (e.g. travel costs); impact on social care services (both publicly and privately funded; community and domiciliary care).

In the UK, the Orthopaedic Data Evaluation Panel (ODEP) [11] provides a rating for prostheses based on data submitted by the manufacturers. For example, the Charnley cemented cup and stem both have a rating of 10A, designating strong clinical evidence of prosthesis survival at 10 years (NICE benchmark) [5]. However, to date, no studies has systematically summarised current economic evidence to compare the impact of different types of prostheses on costs and cost-effectiveness.

The objective of this systematic review is to critically appraise and summarise current published evidence on the costs and cost-effectiveness of using alternative prostheses in THR surgery.

More specifically, we aim to:

1. Assess the completeness of the evidence base for resource use, costs and cost-effectiveness;

2. Assess the applicability of the available evidence to inform resource allocation decisions in the UK NHS.

## Methods

Our search strategy, criteria to identify relevant papers and approach to data extraction are described below.

### Criteria for considering studies for this review

#### Types of studies

**Figure 2:**
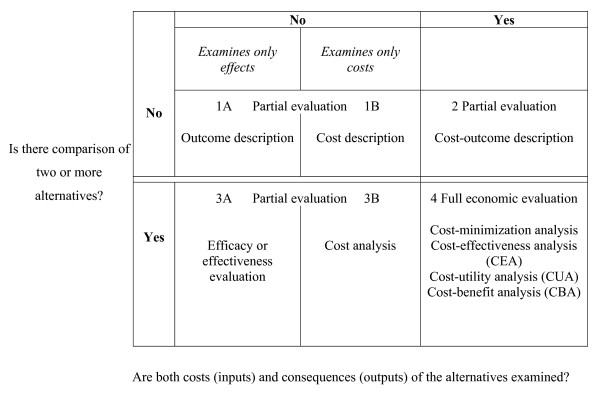
**Distinguishing characteristics of health care evaluation **[12].

Full economic evaluation studies (cost-effectiveness analyses, cost-utility analyses or cost-benefit analysis), defined as the comparative analysis of alternative courses of action (e.g. healthcare treatments) in terms of both their costs and their consequences (e.g. clinical effects) [12]. Partial economic evaluation studies which compare alternatives in terms of their costs only (i.e. cost analyses) [12]. (See figure 2.)

#### Types of participants

Adults 18 years or over.

#### Types of Interventions

Any THR surgery using any type of hip prosthesis (using any surgical technique) compared to THR surgery using any other type of prosthesis (any surgical technique).

#### Types of outcome measures

1. Direct medical resource use; Prosthesis, operative time, post-operative care, length of post-operative hospital stay (los), management of surgical/implant/post-operative complications, medication, use of therapy services, use of adult social care services, revision surgery within follow-up period, long-term revision surgery (prosthesis failure)

2. Non-medical resource use; Productivity losses (sick days, lost wages) - patient: productivity losses (sick days, lost wages) - informal carer(s): other patient/family out-of-pocket expenses (travel to hospital visit)

3. Health effects; Post-operative pain, surgical/implant/post-op complications, physical functioning, health related quality of life (HR-QoL), mortality/survival, quality adjusted life years (QALYs),

Note direct assessments of revision and bilateral surgery are excluded in the review.

### Search methods for identification of studies

#### Electronic searches

We searched MEDLINE (1950 to May 2010); EMBASE (1980 to 2010 week 20) Cinahl (1971 to May 2010); The Cochrane Library (Issue 5, 2010): The Cochrane Database of Systematic Reviews; Database of Abstracts of Reviews of Effects (DARE) and Health Technology Assessment (HTA) database; Health Economic Evaluations Database (HEED) (1992 to 6 June 2010); the NHS Economic Evaluation Database (NHS EED) (1992 to 6 June 2010) and the European Network of Health Economic Evaluation Databases (EURONHEED) (2000 to 6 June 2010).

A search strategy was developed and adapted for use in each electronic database. An example of the search strategy used in OVID Medline is given in 'Additional file 1, Appendix 1'.

#### Searching other resources

Grey literature searching was outside the scope of this review. However, we reviewed bibliographies of the included economic evaluations to identify additional eligible economic evaluations.

### Data collection and analysis

#### Selection of studies

One researcher screened the titles and abstracts of the literature search results for eligible economic evaluations. Full text reports of all eligible studies were sought. Excluded studies were listed with the reasons for their exclusion. Articles published in languages other than English were excluded since translation was outside the scope of the current review.

#### Data extraction and management

One researcher carried out all data extraction using a two-stage process [13]. First, risk-of-bias in generating clinical effect estimates utilised in each economic evaluation (if applicable) was assessed using a tool endorsed by the Cochrane Bone, Muscle and Joint Trauma Group [14]; Study quality was assessed using a more general tool, the Critical Appraisal Skills Programme (CASP) checklist for: (i) cohort studies [15] and (ii) randomised controlled trials [16]. Next, an overall assessment of the methodological quality of each economic evaluation was made, informed by applying the guidelines for authors and peer reviewers of economic submissions to the BMJ and, in the case of model-based full economic evaluations, a checklist for best practice guidelines in decision-analytic modelling [17]. An example of a completed data extraction form is presented in 'Additional file 2, Appendix 2'.

#### Data Synthesis

The extracted data were synthesised by summarising the methodological quality of each study in tables, these tables were then supplemented with a narrative summary. All estimates of costs reported in the literature were converted to British currency values (GBP) using exchange rates based on Purchasing Power Parities and inflated to 2008 prices using a web-based conversion tool [4]. Results are reported according to: study type, perspective, comparator, study design, time horizon, data sources, health benefit measures, discount rate, uncertainty and sponsorship.

## Results

### Description of studies

#### Results of the search

**Figure 3:**
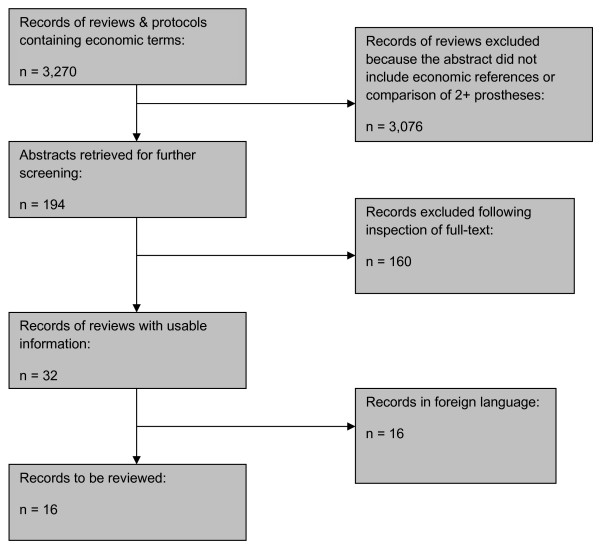
**Quorum statement flow diagram **[13]. Summary of searches.

3,270 papers were retrieved by electronic searches (Figure 3). Of these 194 potentially eligible abstracts were retrieved for further screening. Papers were excluded if they did not compare two or more prostheses or were not a full or partial economic evaluation. 16 studies identified for possible inclusion are not reported in English and in some cases did not include an English language abstract, these studies are not included in this review. A total of 17 potentially eligible studies were identified amongst 194 abstracts and are therefore included in this review.

#### Included studies

Additional File 3, Table S1 provides a summary of included studies based on the Drummond et al checklist for economic evaluation studies [18]. A narrative summary of the characteristics and methods of included studies is presented below.

##### Study Design

Ten studies are classified as full economic evaluations (cost-effectiveness analyses [19-23] and cost-utility analyses [8,24-27]; no eligible cost-benefit analyses were identified). These studies either employ the survival rate of the prosthesis as the measure of health benefit [19-23], or combine survival and HR-QoL measures to calculate QALYs [8,24-27]. Nine studies are model-based evaluations and these can be further classified into two sub-groups: (i) deterministic models (e.g. Daellenbach et al [21]) and (ii) probabilistic Markov model (e.g. Briggs et al [8]).he stated purpose of some of these studies is largely methodological [8,20,21,26]; they aim to develop a methodology which can also be applied to other healthcare interventions, using THR and the specific prostheses as an illustrative example to demonstrate a more widely generalisable modelling approach. However, this fact does not limit the reliability of the findings of these studies. Indeed, results from Briggs et al [8] have been used to inform NICE guidelines on hip prostheses [5]. One CUA is a retrospective cohort study conducted using additional questionnaire data [27].

Seven studies [28-34] are classified as cost analyses. Average total costs per patient by treatment group (surgery or prosthesis type) are the main outcome measures reported in these seven studies.

##### Country

Seven studies were based primarily on UK data, with the others based primarily on data from Australia, USA, Sweden, New Zealand, Germany, Italy Israel and Belgium. Full economic evaluations using revision rates for prostheses derived from populations outside of the UK [8,20,21,27] would need to be further examined for differences in patient characteristics and surgical implantation techniques before results could be applied to the UK setting. Cost analysis studies [28,30,32,34] using data from outside of the UK are based on different health care systems with differing study populations, thus generalisability of these results to the UK setting are of limited use other than to explore cost variation of prostheses as a component of THR surgery. Furthermore, some of the older studies using UK data are limited use in terms of the relevance to current NHS practice [35].

##### Interventions

Only one full economic evaluation conducts a head-to-head comparison between two brands of hip prostheses [8]. Four studies compare the Charnley prosthesis with an unspecified alternative (see Additional file 3, Table S1) and ten studies report the comparison as either ‘cemented vs cementless' or ‘cemented/or hybrid' (see Additional file 3, Table S1), with no brand information. Scheerlink et al [30]make cost comparisons across three different brands of prostheses and an unnamed ‘other'.

##### Time horizon

NICE [10] recommends using a time horizon sufficiently long to reflect all important differences in costs and outcomes between the alternatives under evaluation. In this case, hip prostheses can last for up to approximately 20 years following implantation [11]. As Additional file 3, Table S1 reports, a variety of time horizons are used for model-based economic evaluations included in this review, ranging from five years [25] to 60 years [8,24,26].

##### Analytic perspective

General guidance on conducting an economic evaluation recommends adopting a broad societal analytic perspective as the gold standard, but it is widely recognised that a narrower analytic perspective (e.g. health care system) may be sufficient if the purpose of the evaluation is to inform decisions that will be made within a narrower constituency (e.g. health care system) [18]. All studies identified in this review consider only those costs (resource use) relevant from the perspective of the health care system. One study [21] mentions the wider perspectives of society and the patient but resource use and costs that would be relevant from these perspectives are not included in the analysis.

##### Outcome measures of health gain

Five of the full economic evaluation studies report survival rate of the prosthesis as the primary measure of health benefit; either as an observed rate (see Additional file 3, Table S1), or a rate statistically extrapolated over a longer time horizon (see Additional file 3, Table S1). Three studies [22-24] report survival rates for prosthesis types, varying the length of years through sensitivity analysis of the extrapolated survival rates at which survival was recorded. In general, there is a lack of long-term prosthesis survival data. In order to overcome this difficulty, studies employ statistical extrapolation of prosthesis survival data over a longer time horizon. Briggs et al [8] examine a range of parametric survival models and conclude that the Weibull distribution fits best to the data; the data are then extrapolated over 60 years.

While survival is a useful measure of health gain, QALYs have the advantage that they combine length of survival with quality of life. Thus they enable comparisons between different health-care interventions in terms of a single measure of relative efficiency (i.e. cost per QALY), informing resource allocation decisions based on considerations of allocative efficiency across interventions [36]. Five economic evaluation studies used QALYs as their composite measure of health benefit [8,24-27]. However, only Briggs et al [8] and Givon et al [27] conducted primary research on HR-QoL in a THR patient population to inform QALY estimates. Briggs et al used the EQ-5D questionnaire and Givon et al used the Rosser index to inform QALY estimates.

### Direct medical resource use, unit costs and costs

Table 1 records the unit costs of the prostheses reported in each study: it shows the range between the cheapest and most expensive for the two broad types of prosthesis, and then for specific named prostheses within each type. In general, cemented prostheses were cheaper than cementless, ranging (in the literature) from £691 (Multicentre) [33] to £2,845 (Beuchel Pappes) [33] for cementless, compared with £455 (Stanmore) [33] and £1,693 (Titan) [33] for cemented.

**Table 1:** Prosthesis costs (inflated to 2008 prices, in GBP) [4]

	Min cost prosthesis (literature)	Max cost prosthesis (literature)
**CEMENTED (Mean)**	£515 [21]	£1,084 [30]
**Charnley**	£395 [8]	£943 [29]
**Stanmore**	£455 [33]	£990 [29]
**Titan**	£1,693 [33]	£1,693 [33]
**CEMENTLESS (mean)**	£1,819 [31]	£5,785 [34]
**Multicentre**	£691 [33]	£960 [33]
**Spectron**	£903 [8]	£1,134 [22]
**Buechel Pappes**	£2,845 [33]	£2,845 [33]
**HYBRID (mean)**	£1,886 [32]	£4,452 [34]

The average total cost of the THR procedure per patient reported in the studies ranges from £4,599 [23] to £8,078 [30]. Most studies reporting resource use and costs with the cost of the prosthesis assume these to be equal for each prosthesis type [33].

According to Scheerlink et al [30] implantation of the prosthesis (including the prosthesis itself), accounts for the second largest component of the total cost of THR surgery (21.3%), with hospital length of stay (LOS) being the largest component. The reported range of mean LOS in days is from 7.3 [33] to 23 [31] with meancosts varying from £2,101 [23]to approximately £7,081 [22] (obtained through sensitivity analysis).

The range for duration of surgery (theatre time) is 60 to 246 minutes [30]. Unnanuntana [28] is the only study to report duration of surgery separately for cemented, cementless and hybrid (femoral stem), finding that operative time for a cementless stem is approximately 20 minutes less than for both hybrid and cemented stems. Reported costs for duration of surgery show wide variation from £1,128 [24] to £6,176 (obtained through sensitivity analysis) [22]. Scheerlink et al [30] reports medication costs as approximately 9% of the total cost of the procedure, breaking them down according to prosthesis brand, but reporting no apparent differences.

#### Non-medical resource use

No studies report non-medical resource use.

#### Data sources used to populate the model

Nine studies used primary research to inform their analysis (for example, as discussed above Briggs et al elicited HR-QoL data from THR patients) with the remaining eight all using purely secondary data sources.

#### Sensitivity analysis

Only one of the full economic evaluation studies [27] does not conduct sensitivity analysis to address uncertainty. In their 2009 guidance, NICE describe three types of potential selection bias or uncertainty to consider: Structural uncertainty (categorisation of different states of health and the representation of different pathways of care); source of values to inform parameter and parameter precision (uncertainty around the mean health and cost inputs in the model).

Daellenbach et al [21] perform sensitivity analysis on the ‘less-reliable' input data defined as: the intangible costs of re-operation surgery (implicitly including those of the patient) and the expected failure rate of the prosthesis. Baxter and Bevan [22] perform sensitivity analysis on many of the parameters of their model, identifying the main cost drivers (hospital costs, prosthesis price and revision rates). Gillespie et al [20] conduct sensitivity analysis on the ‘break-even price ratios' for hypothetical prostheses at various years using four hypothetical rates of prosthetic failure. Briggs et al [8] and Spiegelhalter and Best [26] use probabilistic sensitivity analysis (PSA) applied to parameter uncertainty in the model, conducting sub-group analysis by age and gender. Marinelli et al [25] also perform sensitivity analysis on revision rates, prosthesis costs, preoperative mortality, infection rates and utility values, however the details of the approach employed are not fully reported.

### Risk of bias

The reliability of any full economic evaluation depends in part on its use of reliable clinical data, including data on beneficial and adverse effects, complications and secondary interventions [13]. Most of the included studies use observational data, such as from joint registries, to inform their analysis. Although RCTs are often thought of as the gold standard to inform economic evaluation studies [37], evaluation of THR is a context where the use of RCTs is of limited use in terms of the nature of the procedure - the long-term follow-up to observe time until revision surgery. Additional file 4, Table S2 reports the outcomes for risk of bias. No studies report blinding or randomisation due to the type of studies included in this review. Additional file 4, Table S2 shows that of the seventeen studies, inclusion and exclusion criteria is stated in five studies, and the intervention and outcome measures are defined in thirteen and fourteen respectively.

#### Discount Rate

All but one [27] of the full economic evaluation studies use a discount rate to account for time preference of costs and benefits which accrue in the future, varying from 5 to 6% for costs and 1.5 to 6% for benefits.

### Summary of main results

#### Incremental Cost Effectiveness Ratios (ICERs)

Table 2 reports the ICERs for the three economic evaluations studies who report ICERs [8,25,26] (the extra cost per unit of outcome obtained, in comparing one treatment with another) [38]. It is important to note here that the limited reporting of the methods for Marinelli et al [25] makes the strength of their findings difficult to assess and that Speigelhalter and Best [26] state their results should “not be taken as contributing in any way to guidance as to an appropriate prosthesis” (pg 3692). The remaining 13 studies do not report ICERs as they do not include a HR-QoL outcome in their study.

**Table 2:** Incremental Cost-Effectiveness Ratios (ICERs)*

Study	Males	Females
**Briggs (2004)**	80 years £946/QALY	70 years £829/QALY
	90 years £14,408/QALY	80 years £8,622/QALY
		90 years £20,742/QALY
**Marinelli (2008)**	Cementless prosthesis £48	
Spiegelhalter (2003)**	55-64 years £739/QALY	55-64 years £683/QALY
	65-74 years £6,604/QALY	65-74 years £5,993/QALY
	75-84 years £16,823/QALY	75-84 years £153,090/QALY
	greater than 84 years £27,780/QALY	greater than 84 years £23,912/QALY

*Costs inflated to 2008 prices, in GBP [4]

** illustrative only, authors state results should “not be taken as contributing in any way to guidance as to an appropriate prosthesis” [26]

#### Other Results

Table 3 shows the cost per QALY gained for baseline cases reported in Givon et al [27]. They find that the cut off point where a hydroxyapatite coating (HA) implant becomes cost-effective is at a baseline QALY of 0.74 compared to all alternatives. Daellenbach at el [21] conclude that the higher cost cementless prostheses must last 6 to 9 extra years before revision surgery in order to yield the same expected present value as a cemented prosthesis. Fitzpatrick et al [24] report that of the cemented prostheses, the Charnley, Stanmore and Exeter perform relatively well in terms of time until prosthesis failure. Based on their model, they report that a cementless prosthesis costing approximately 300% more than the Charnley or other established prostheses was unlikely to reduce the revision risk sufficiently to produce any cost savings. Two studies [22,23] report results for the Stanmore and Charnley by calculating the total expected cost of the prostheses over 20 years, reporting that the Stanmore is slightly more cost-effective than the Charnley.

**Table 3:** Cost per QALY*

Study	Cementless	Cemented	Hybrid	HA-coated
**Givon 1998**	0.50/£10241	0.50/£7749	0.50/£10352	0.50/£9728
	0.60/£13108	0.60/£10329	0.60/£13290	0.60/£12279
	0.70/£18203	0.70/£15484	0.70/£18556	0.70/£16643
	0.80/£29775	0.80/£30732	0.80/£30732	0.80/£25815

*Costs inflated to 2008 prices, in GBP [4]

## Discussion

This paper has systematically searched for, assessed and summarised literature on the costs and cost-effectiveness of using alternative prostheses in THR surgery. We have identified several methodological problems in the literature including a lack of observed long term prosthesis survival data, limited up-to-date UK based evidence and exclusion of patient and societal perspectives.

Several limitations of this systematic review should be highlighted when interpreting these principal findings. Foreign language studies were considered outside the scope of this review, thus sixteen studies were excluded. For all foreign language studies, English language abstracts were sought to further determine whether the study met the inclusion criteria, in some cases no abstract at all or no English language abstract was available. In the remaining cases it was not clear from the abstract whether or not the study would meet the inclusion criteria. From screening titles, all foreign language studies appear to be partial economic evaluations and thus the generalisability of the study to the UK context (for the purpose of this review) is anticipated to be limited due to international differences in health care settings.

Hand searches and grey literature searches were not undertaken. Literature searching, data extraction and critical appraisal were carried out by the first author only. Inclusion of a further assessor would have reduced the risk of bias in study selection and the risk of error in data collection.

Only seven studies were based primarily on UK data with some of the older studies being of limited use in terms of the relevance to current NHS practice. Where studies were non-UK based, revision rates for prostheses derived from populations outside of the UK require further detail of patient characteristics and surgical implantation techniques before results can be applied to the UK setting. Cost analysis studies have generally been based on different health care systems with differing study populations, thus limiting the applicability of these results to the UK, NHS context.

One of the methodological limitations of the studies identified in this review is the different types of economic models used, making comparability across results difficult: none of the studies compared alternative models to answer the same question. The main difference between the types of model identified in this review is the description of disease progression. Markov modelling [8,24-26] involves dividing a patient's possible prognoses into a series of health states. The probabilities defining the transitions between each of these states are specified over a single cycle of the model [24]. The model is then run over a number of cycles to view how a typical patient would move between states over a specified time period, consisting of several cycles. The transition probabilities reported in the Markov models in this review are calculated based on data obtained from a range of different sources, including life tables, clinical trials and other published sources. Crucially, because the empirical studies typically observe data used to generate transition probabilities over a limited follow-up period, the authors also employ statistical methods to extrapolate beyond the time horizon of observed data, for example the risk of revision. The Markov models identified in this review, are also fully probabilistic in their approach to managing uncertainty in the model parameters, NICE now requires the use of PSA for all cost effectiveness submissions [10].

The deterministic cost-effectiveness models (Daellenbach et al) [21] use more simplified assumptions. A key difference relates to the treatment of prosthesis survival rates. While studies using a Markov approach allow for the possibility that a prosthesis may fail at any point in time (according to a probability distribution), deterministic models assume a range of values for the expected life of a cemented prosthesis and then determine, for each of these values, the increase in the expected life of a cementless prosthesis required in order for the two to have the same net present value cost (for various age groups). This assumes that a prosthesis will fail at a specific point in time. Other studies[19,20,22,23] use a similar approach. Faulkner et al [23] estimate expected costs over twenty years using data from other studies and using statistical extrapolation to predict future revision rates.

A significant knowledge gap and challenge to research in this area relates to observed survival rates. NICE currently define their benchmark for revision rate as being 10% at 10 years [5]. Some studies in this review have employed methods of extrapolation of the data in order to estimate survival rates into the future. However, these are based on very short time periods of observed data. This highlights the need for more trials comparing different prostheses with long-term follow up. Only one full economic evaluation carried out a head-to-head comparison between two different manufacturer named prostheses [8]. Further economic evaluations of the prostheses according to their manufacturer rather than type (cemented/cementless) are needed given the large number of prostheses, the likely variability within specific types of prostheses and the technological changes that have occurred over time. It is recommended that clinical trials should include an economic evaluation during pre-trial modelling (employing a Bayesian iterative approach), which would inform the trial design and subsequent extrapolation of trial data [39].

In order to comprehensively assess whether an intervention provides value-for-money, information on non-medical resource use and productivity losses should be sought and taken into account, even though not required in assessment guidelines for some agencies (e.g. NICE). Failure to take into account these costs and benefits may hide the fact that they are being merely shifted onto another sector [40]. We have identified very limited consideration of the patients' and society's costs and resource use in the literature. Baxter and Bevan [22] recommend further research combining prosthesis survival and HR-QoL.

This review also highlights the lack of up-to-date published studies using UK data, fourteen out of the seventeen studies included in this review were conducted over five years ago. The recent development of the NJR may provide an opportunity to produce more up-to-date analysis using data from England and Wales.

Finally, the range of costs of prostheses from Additional file 3, Table S1 provides an interesting perspective regarding the NHS national tariff for primary THR (an individual tariff is derived for each hospital patient episode, represented by the average cost of providing a particular procedure) [41]. This tariff specifies how much hospitals are reimbursed for treatments, in 2008/9 this was £5,220 for cemented and £5,587 for cementless prostheses (2008/9) [42]. The tariffs include a component for length of stay (currently £4,262 and £4,193 respectively) [42], implying very low tariffs for the surgical procedure itself (about £1,000 and £1,400 respectively). This is deserving of further research, to understand the potential tradeoffs that could occur across the range of prostheses in terms of ‘profit' versus effectiveness.

## Conclusions

There is a need for more clinical trials including economic evaluations [43] and comparing different prostheses with long-term follow up. These trials should also consider the perspectives of the health service, patients' and society. The recent development of the NJR (England and Wales) provides a unique opportunity for international comparisons of those countries with existing joint registries and to address the gap in the literature on the cost effectiveness of hip prostheses in England and Wales.

## Competing interests

The authors declare that they have no competing interests.

## Authors' contributions

CD designed the review, synthesized and analysed the data and wrote the manuscript. IS contributed to defining the research question and search strategy. PL contributed to the analysis, formulating results and writing of the manuscript. MM, KT and AM contributed in editing the manuscript. All authors have read and approved the final manuscript.

## Supplementary Material

Additional file 1 **Appendix 1**. Search strategy for OVID Medline. Click here for file

Additional file 2 **Appendix 2**. Example data extraction form. Click here for file

Additional file 3 **Table S1**. Summary of economic studies comparing hip prostheses Excel Table reporting key findings from the critical appraisal of included studies. Click here for file

Additional file 4 **Table S2**. Risk of Bias in effectiveness evidence Excel table reporting the risk of bias evaluation of the studies. Click here for file
