# Supplementary material for: Can choices between alternative hip prostheses be evidence based? a review of the economic evaluation literature
Source: Cost Eff Resour Alloc. 2010 Oct 29;8:20. doi: 10.1186/1478-7547-8-20 (PMC2984411; doi:10.1186/1478-7547-8-20)
Supplement: Additional file 1 — Appendix 1. Search strategy for OVID Medline. [file 1478-7547-8-20-S1.DOC]

**Appendix 1 : Search strategy for OVID Medline (updated search: May 2010**)

| **Searches** | **Results** |
| --- | --- |
| total hip replacement.mp. | 5164 |
| *Arthroplasty, Replacement, Hip/ | 9477 |
| total hip arthroplasty.mp. | 7298 |
| (hip adj prosthes$).tw. | 2776 |
| or/1-4 | 18040 |
| cost$.mp. | 325088 |
| resource use$.mp. | 3066 |
| *Cost-Benefit Analysis/ | 3539 |
| *"Costs and Cost Analysis"/ | 4832 |
| *Economics/ | 9922 |
| *Models, Economics/ | 0 |
| economic evaluation$.mp. | 4375 |
| *Economics, Medical/ | 4996 |
| or/6-13 | 339390 |
| 5 and 14 | 745 |
