# Supplementary material for: Can choices between alternative hip prostheses be evidence based? a review of the economic evaluation literature
Source: Cost Eff Resour Alloc. 2010 Oct 29;8:20. doi: 10.1186/1478-7547-8-20 (PMC2984411; doi:10.1186/1478-7547-8-20)
Supplement: Additional file 2 — Appendix 2. Example data extraction form. [file 1478-7547-8-20-S2.DOC]

**Appendix 2 – Example data extraction form (based on Drummond et al checklist [17]** for economic evaluations)

| **Study name: Marinelli ,2008** | |  |  |  |  |  |
| --- | --- | --- | --- | --- | --- | --- |
| **Item** | | **Yes** | **No** | **N/C** | **N/A** | **Extract/ comments** |
| **Study design.** | |  |  |  |  |  |
| 1. | The research question is stated. | 🗸 |  |  |  | “To establish a framework in which to evaluate the cost-effectiveness of cementless and cemented implants and to analyse how device cost and revision affect the model” |
| 2. | The economic importance of the research question is stated. | 🗸 |  |  |  | “Randomized controlled trials are the gold standard for demonstrating the clinical benefits of new technologies. However, detecting small differences in failure rates among implants requires randomizing large numbers of patients and following them for extended periods (15-20 years, or longer). These studies are difficult to perform due to practical considerations of time and cost. In contrast, decision-analysis techniques offer the potential to analyze the performance of a new technology prior to the availability of long-term clinical outcome data. Furthermore, the results from a well-designed decision analysis study can guide further clinical and laboratory research based on the variables that the have the greatest influence on cost-effectiveness. Finally, a cost effectiveness framework can also be readily updated as new information on cost and clinical effectiveness emerges from randomized trials and cohort studies.” |
| 3. | The viewpoint(s) of the analysis are clearly stated and justified. | 🗸 |  |  |  | “The costs of cementless and cemented THA were estimated from a payer perspective using average hospital costs for prosthetic implants in 2006 Euro’s” |
| 4. | The rationale for choosing alternative programmes or interventions compared is stated. | 🗸 |  |  |  | Cemented and cementless implants – this reflects current standard practice. |
| 5. | The alternatives being compared are clearly described. |  |  | 🗸 |  | “Several different devices (..) are regularly implanted at our Orthopaedics Department. “ |
| 6. | The form of economic evaluation used is stated. | 🗸 |  |  |  | The authors classify the study as cost-effectiveness analysis. Using the Drummond checklist, it could be described as a cost-utility analysis. |
| 7. | The choice of form of economic evaluation is justified in relation to the questions addressed. |  |  | 🗸 |  | “A Markov decision model was used to analyze a theoretical cohort of 70-year patients….” |
| **Data collection.** | |  |  |  |  |  |
| 8. | The source(s) of effectiveness estimates used are stated. | 🗸 |  |  |  | Data on prosthesis revision rates is taken from a prosthesis register (RIPO register). Age-specific probability of death was determined from 2001 United States Life Tables. Published sources were used for other clinical estimates such as peri-operative death and utilities in the model were based on index scored reported in the literature. Methods used to derive the estimates was not explicit. |
| 9. | Details of the design and results of effectiveness study are given (if based on a single study). |  |  | 🗸 |  | Further details on the study methodology and greater detail on deriving effectiveness sources is required. |
| 10. | Details of the methods of synthesis or meta-analysis of estimates are given (if based on a synthesis of a number of effectiveness studies). |  |  | 🗸 |  | Further details on the methods of synthesis is required. |
| 11. | The primary outcome measure(s) for the economic evaluation are clearly stated. | 🗸 |  |  |  | Quality Adjusted Life Years (QALYs) discounted at a yearly rate of 3%. QALYs were estimated using the Markov model. |
| 12. | Methods to value benefits are stated. | 🗸 |  |  |  | Utilities were based on quality well-being index scores reported in the literature. |
| 13. | Details of the subjects from whom valuations were obtained were given. |  |  | 🗸 |  | Information on utility scores provided but not on subject details other than age. |
| 14. | Productivity changes (if included) are reported separately. |  | 🗸 |  |  | Not discussed |
| 15. | The relevance of productivity changes to the study question is discussed. |  | 🗸 |  |  | The authors acknowledge that lack of inclusion of all societal costs is a limitation of the study. |
| 16. | Quantities of resource use are reported separately from their unit costs. |  |  | 🗸 |  | Resource use not reported in detail or source |
| 17. | Methods for the estimation of quantities and unit costs are described. |  |  | 🗸 |  | Yes, but only prosthesis cost. Costs were not broken down. |
| 18. | Currency and price data are recorded. | 🗸 |  |  |  | Euro 2006 |
| 19. | Details of currency of price adjustments for inflation or currency conversion are given. |  |  |  | 🗸 | n.a |
| 20. | Details of any model used are given. | 🗸 |  |  |  | Markov model was used. The model structure was provided in a figure. |
| 21. | The choice of model used and the key parameters on which it is based are justified. | 🗸 |  |  |  | Appropriate choice of model for this setting. |
| **Analysis and interpretation of results** | |  |  |  |  |  |
| 22. | Time horizon of costs and benefits is stated. |  |  | 🗸 |  | Not made explicit, although it appears to be 5 years. |
| 23. | The discount rate(s) is stated. | 🗸 |  |  |  | 3% applied to costs and outcomes. |
| 24. | The choice of discount rate(s) is justified. | 🗸 |  |  |  | Reference for choice provided. |
| 25. | An explanation is given if costs and benefits are not discounted. |  | 🗸 |  |  | n.a |
| 26. | Details of statistical tests and confidence intervals are given for stochastic data. |  |  | 🗸 |  | The model is reportedly probabilistic, although details of this in the methodology and results is not provided. |
| 27. | The approach to sensitivity analysis is given. |  |  | 🗸 |  | A sensitivity analysis was performed on revision rates, prosthesis costs, preoperative mortality, infection rates and utility values. Details of the sensitivity analysis is not fully reported and thus not fully justified. |
| 28. | The choice of variables for sensitivity analysis is justified. |  |  | 🗸 |  | See no. 27 |
| 29. | The ranges over which the variables are varied are justified. |  |  | 🗸 |  | See no.27 |
| 30. | Relevant alternatives are compared. | 🗸 |  |  |  | See sections 4 & 5 |
| 31. | Incremental analysis is reported. | 🗸 |  |  |  | Yes |
| 32. | Major outcomes are presented in a disaggregated as well as aggregated form. |  | 🗸 |  |  | Outcomes are only reported in aggregated form. |
| 33. | The answer to the study question is given. | 🗸 |  |  |  | The authors conclude that the risk of revision is similar between cemented and cementless prosthesis groups, in terms of QALYs and the cost-difference as non-significant. |
| 34. | Conclusions follow from the data reported. |  |  | 🗸 |  | Conclusions follow. However, the conclusions are hard to follow due to the limited reporting of methodology, sources and presentation of results. |
| 35. | Conclusions are accompanied by the appropriate caveats. |  |  | 🗸 |  | Further clarity could be provided. |
